# A study on the tolerance level of farmers toward human-wildlife conflict in the forest buffer zones of Tamil Nadu

**DOI:** 10.14202/vetworld.2016.747-752

**Published:** 2016-07-22

**Authors:** K. Senthilkumar, P. Mathialagan, C. Manivannan, M. G. Jayathangaraj, S. Gomathinayagam

**Affiliations:** 1 Department of Wildlife Science, Madras Veterinary College, Tamil Nadu Veterinary and Animal Sciences University, Chennai, Tamil Nadu, India; 2 Department of Veterinary and Animal Husbandry Extension, Madras Veterinary College, Tamil Nadu Veterinary and Animal Sciences University, Chennai, Tamil Nadu, India; 3 University Publication Division, Directorate of Distance Education, Tamil Nadu Veterinary and Animal Sciences University, Madhavaram Milk Colony, Chennai, Tamil Nadu, India; 4 Department of Veterinary Parasitology, Madras Veterinary College, Tamil Nadu Veterinary and Animal Sciences University, Chennai, Tamil Nadu, India

**Keywords:** elephant, gaur, human-wildlife conflict, local perceptions, monkey, tolerance level, wild pig

## Abstract

**Aim:**

The aim of this work was to study the tolerance level of farmers toward different human-wildlife conflict (HWC) situations.

**Materials and Methods:**

This study was conducted in 24 villages of nine blocks from Kancheepuram, Coimbatore, Erode, and Krishnagiri districts of Tamil Nadu by personally interviewing 240 farmers affected with four different HWC situations such as human-elephant conflict (HEC), human-wild pig conflict (HPC), human-gaur conflict (HGC), and human-monkey conflict (HMC). A scale developed for this purpose was used to find out the tolerance level of the farmers.

**Results:**

In general, the majority (61.70%) of the farmers had medium level of tolerance toward HWC, whereas 25.40% and 12.90% belonged to a high and low category, respectively. The mean tolerance level of the farmer’s encountering HMC is low (8.77) among the other three wild animal conflicts. In tackling HWC, the majority (55.00%) of the HEC farmers drove the elephant once it entered into their farmland. In the HPC, more than three-fourths of the respondents drove away the wild pig once they were found in farmlands. With regard to the HMC, a less number of them (1.70%) drove the monkey away if monkeys were spotted in their village. With regard to HGC, 95.00% of the respondents frightened the gaurs if their family members were threatened by gaurs.

**Conclusion:**

The present study suggests that that majority of the farmers had medium level of tolerance toward HWC. The tolerance level of the HMC farmers was lower than other three HWC affected farmers. This study emphasizes the need for necessary training to tackle the problem in an effective manner for wild animal conservation.

## Introduction

Forests in Tamil Nadu occupy 22,877 km^2^, which is 17.59% of the State’s geographical area [1]. To undertake complementary activities of biodiversity conservation and development of sustainable management, biosphere reserves are demarcated into three inter-related zones, *viz*., natural or core zone, manipulation or buffer zone, and a transition zone outside the buffer zone [2]. A buffer (safety) zone of 2 km, for country’s national parks and wildlife sanctuaries having an area of 200 km^2^ or more, is mandatory. Each pocket of wildlife habitat can be viewed as an island surrounded by human settlements.

Human-wildlife conflict (HWC) occurs when wildlife requirements encroach on those of human populations, with costs both to residents and wild animals. HWC occurs due to various reasons that include human population growth rate, the increasing demand for natural resources, and the growing pressure for access to land.

A proper understanding of the tolerance level of individual wild animal affected farmers paves a way for the rational design for the effective preventive and control measures of HWC. In addition, the results of this study help the forest official to take proper policy decision for theconservation of HWC creating animals. Although most of the studies have been carried out with respect to economic losses due to human-elephant conflict (HEC) [3], perception and possible solutions of HWC [4,5], and methods used to mitigate HEC [6], there is no much study on tolerance level on HWC; hence, the present study was undertaken to assess the tolerance level among the farmers affected with different HWC situations.

## Materials and Methods

### Ethical approval

This study was meant to elucidate the tolerance level of farmers towards human-wildlife conflict through personal interview and it neither involved any animal experiments nor handling of wildlife, clearance from the Animal Ethical and Bio-safety Committee was not warranted.

### Locale of the study

The study was carried out in Coimbatore [human-elephant Conflict (HEC)], Krishnagiri (human-wild pig conflict [HPC]), Erode (human-gaur conflict [HGC]), and Kancheepuram (human-monkey conflict [HMC]) districts of Tamil Nadu state, India.

### Methods of sampling

#### Selection of district

Among the 32 districts of Tamil Nadu states, the study was purposively carried out in Erode (HGC), Coimbatore (HEC), Krishnagiri (HPC), and Kancheepuram (HMC) district of Tamil Nadu state due to the high incidence of HWC in these districts on the basis of data collected from Tamil Nadu Forest Department.

#### Selection of blocks and villages

HGC district

In Erode district, four blocks were within the forest buffer zones. From these four blocks, two blocks (Thoockanaickenpalaiyam and Sathyamangalam) were selected by simple random sampling technique. From each of the two blocks selected, three villages were selected by simple random sampling technique. Thus, a total of six villages were selected.

HEC district

Out of six blocks in the forest buffer zones of Coimbatore district, three blocks *viz*., Anamalai, Karamadai, and Periyanaickenpalayam were selected by simple random sampling. Then, two villages were selected from each block by simple random sampling. Thus, a total of six villages were selected.

HPC district

From the four forest zone blocks of Krishnagiri district, two blocks, *viz*., Denkanikottai and Thally were selected on simple random sampling. From these two blocks, six villages were also chosen on simple random sampling.

HMC district

A total of two blocks were selected from the four blocks existed in the forest buffer zones of Kancheepuram district by simple random sampling. From the selected bocks, three villages each were selected by applying simple random sampling technique leading to a total of six villages.

From the nine selected blocks, 24 villages were selected by adopting simple random technique.

#### Selection of the respondents

Farmers, who had at least one wildlife conflict incidence in their lifetime, were selected for the study. 60 farmers were selected randomly from each district; thus, a total of 240 farmers were selected from four districts for the study.

#### Tools and techniques of data collection

**Figure-1:**
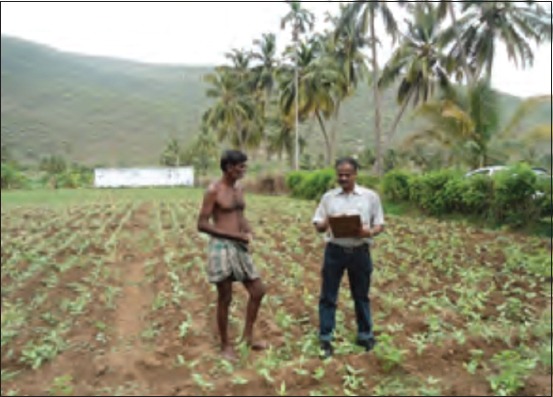
Data collection from human-wildlife conflict victim.

The basic instrument used for the study was the interview schedule (Figure-1). The questions were related to different methods used to drive away the wild animals intruding their farmlands or residences. A total of four statements, which determined their level of tolerance, were spread among four groups of the farmers. If a respondent performs any one of the following activities, *viz*., no action (idle), run away, inform the neighbor, and frighten away, it will fetch a score of 1-4, respectively. Thus, a farmer obtained high score indicated less tolerance level regarding the intrusion of wild animals.

### Statistical analysis

The statistical tools, *viz*., average, percentage, and ANOVA were applied to analyze and interpret the data. Data on the 4 different treatments were analyzed statistically by one-way ANOVA to determine whether a significant difference existed between the 4 different conflicts.

## Results and Discussion

Crop raiding by wild animals is increasingly known to cause conflict between these animals and humans; subsequent losses incurred by farmers might make communities antagonistic and intolerant toward wildlife protection.

Demand of compensation for crop damage, loss of property, and human killing is the very first reaction to the human tolerance [7].

### Distribution of respondents according to the tolerance toward HWC

The respondents were classified into low, medium, and high categories based on the range of score obtained for the tolerance level. The distribution of farmers based on their tolerance level is presented in Table-1.

**Table-1:** Level of tolerance toward HWC situations (n=240).

Category	Frequency (%)
Low	31 (12.9)
Medium	148 (61.7)
High	61 (25.4)
Total	240 (100.0)

HWC=Human-wildlife conflict

The results showed that majority (61.7%) of the farmers had medium level of tolerance toward HWC, whereas 25.4% and 12.9% belonged to a high and low category, respectively. The medium level of tolerance toward HWC showed that presently farmers are more concerned over their own livelihood and not interested toward the forest conservation efforts. Similar findings were reported by many authors [8-10].

### Analysis of distribution of respondents based on tolerance level

Understanding of local tolerance level on the emerging conflicts between people and wildlife was not documented earlier. Only the local people who are living with these kinds of problems can really understand the problem. Further more, gaining this understanding, and acting on such information, is the first step necessary to reduce the emerging conflicts between people and wild animals. Most farmers weretolerant of wild animals that were not perceived as adirect threat.

A detailed analysis on the tolerance level of various groups, *viz*., HEC, HPC, HMC, and HGC of respondents is shown in Table-2.

**Table-2:** Distribution of respondents according to tolerance level toward HWC.

Items	No action				Run away				Inform the neighbor				Frighten away			
			
	E	P	M	G	E	P	M	G	E	P	M	G	E	P	M	G
A wild animal was in your farmland	-	-	-	-	26 (43.3)	3 (5.0)	22 (36.7)	12 (20.0)	1 (1.7)	11 (18.3)	1 (1.7)	3 (5.0)	33 (55.0)	46 (76.7)	37 (61.7)	45 (75.0)
A wild animal was in your neighbors farmland	-	-	10 (16.7)	-	4 (6.7)	-	2 (3.3)	-	42 (70.0)	31 (51.7)	36 (60.0)	31 (51.7)	14 (23.3)	29 (48.3)	12 (20.0)	29 (48.3)
A wild animal was in your own village	14 (23.3)	29 (48.3)	12 (20.0)	29 (48.3)	4 (6.7)	-	-	-	42 (70.0)	31 (51.7)	49 (81.7)	32 (53.3)	14 (23.3)	29 (48.3)	1 (1.7)	22 (36.7)
Your family members felt threatened by a wild animal	-	-	-	-	2 (3.3)	-	-	-	1 (1.7)	11 (18.3)	-	3 (5.0)	57 (95.0)	49 (81.7)	60 (100.0)	57 (95.0)

HWC=Human-wildlife conflict, E=Elephant, P=Pig, M=Monkey, G=Gaur. Figures in parentheses indicate percentage

### Tolerance level of HEC

As far as HEC is concerned, the majority (55.0%) of the HEC farmers drove the elephant once it entered into their farmland (Table-2). Similar findings were recorded at Western Ghats protected areas [11]. Similarly, when their family members were threatened, 95.0% of the farmers drove the elephant from the human habitant irrespective of their age [11].

**Figure-2:**
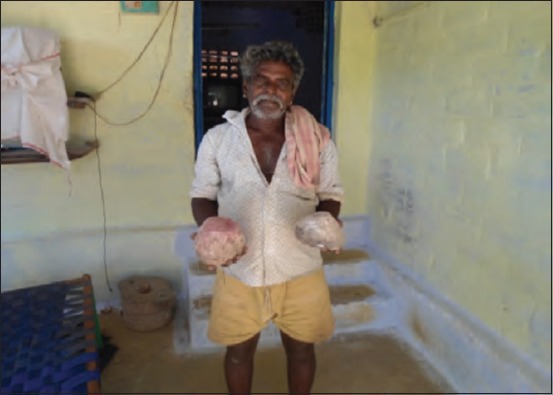
Worshiping elephant’s pug mark.

During field survey, it was observed that some of the farmers believed that elephants stepping into their agricultural field would fetch more profit in near future. So, they lifted the pug mark of the intruded elephant and engraved it as stone carving (Figure-2). They worshiped the pug mark during full moon day of every month. On the contrary, more poaching in the HEC areas was recorded in other study [12].

### Tolerance level of HPC

**Figure-3:**
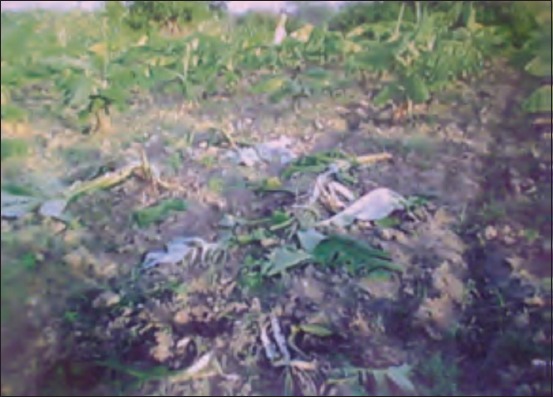
Wild pig trampled field.

In contrary to the HEC, HPC was considered as most burning issue. From Table-2, it could be seen that more than three-fourths of the respondent drove away the wild pig once they found wild pigs in their farmland. This might be due to the fact that the wild pigs damaged the field extensively (Figure-3). Moreover, the respondents used different traditional methods to drive away the wild pigs. This included wire fences with white, flying, flashing ribbons, or plastic strips that produce scaring sounds, and other frightening devices were used in and around crop fields [13,14].

It could further be observed from the Table-2 that 51.70% of the respondents informed the neighbors once they spotted a wild pig in their neighbor’s land. Nearly one-half of the respondents performed no action even when they saw wild pig in their village.

### Tolerance level of HMC

**Figure-4:**
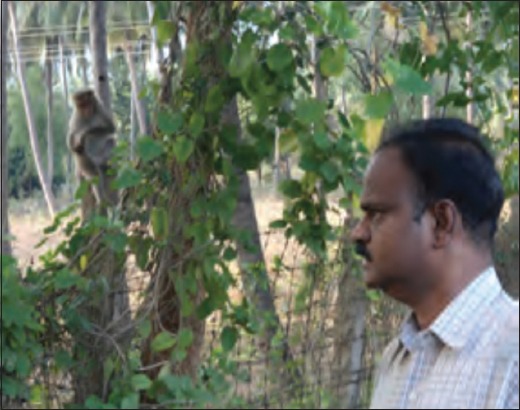
Monkey damaged fencing.

Although HPC created more damage to farmers, monkeys also developed conflict with human beings by damaging the fruit plants, coconut trees, ripened papayas, and plantain crops in the study area. A kind perusal of the Table-2 indicated the tolerance level of the farmers toward monkeys. More than 81.70% of the respondents informed the neighbor if they saw a monkey in their village (Figure-4). In contrary, a meager (1.70%) of them drove away the monkey if they saw the monkey in their village. Discussions with these farmers revealed that they enjoyed seeing monkeys, as was the case with farmers experiencing HEC in Kenya [9]. However, it is surprising to note from the Table-2 that all the respondents were driving the monkey with various methods if it frightened their family members. Farmers in the study area were generally tolerant of monkeys if they had no direct threat to their crops. However, such tolerance did not extend to situations where family members were threatened. Nevertheless, even in these situations, farmers still generally supported the conservation of monkeys.

The Hindu belief in the sacredness of all life and the worshiping of monkeys into ancient Hindu mythology [15], and literature had helped to create a climate of tolerance toward HMC. However, frequent such conflicts with monkeys would affect the traditional bond between man and monkey in near future [16].

### Tolerance level of HGC

As far as tolerance level of HGC, it could be observed from the Table-2 that 95.0% of the respondents frighten away the gaurs if their family members felt threatened by gaur followed by if they saw gaur in their farmland (75.0%), if the animal was in neighbors farmland (48.3%), and if the animal was in their own village (76.7%). The same findings were also recorded [10]. Similarly, 53.3% and 51.7% of the respondents would inform the neighbors when they spotted gaur in their own village and if they noticed the wild animal in their neighbor’s farm land, respectively. Similar situation was found at buffer zones of protected area of Mookambika Wildlife Sanctuary, Kollur, Karnataka [17].

### Relationship between different human-wild animal conflicts and overall tolerance level

The relationship between various human-wild animal conflicts, and overall tolerance level was also studied. It is evident from the Table-3 that the mean tolerance level of the farmer’s encountering monkey conflict was low (8.77) among the other three wild animal conflicts. This revealed that the farmers were accepting the monkeys presence in their neighborhood, in other words, they tolerated the HMC. This was followed by the farmers encountering elephant conflict (9.37), gaur (10.15), and wild pig conflict (10.50). To strengthen this finding, a one-way ANOVA was employed, and the results showed that there was a significant difference in the mean score of tolerance level of adifferent group of farmers.

**Table-3:** ANOVA for significant difference among different HWC with respect todimension of tolerance level of respondents.

Characteristics of respondent		HWC				F value	p value

		Elephant	Wild pig	Monkey	Gaur		
Tolerance level	Mean±SD	9.37±1.966	10.50±1.546	8.77±2.078	10.15±1.885	10.335	<0.001**

** Significant at 1% level. HWC=Human-wildlife conflict, SD=Standard deviation

The main reason for considering the monkeys as high tolerant species was that monkeys were “used to be humans” and were “not bad crop raiders” [18]. The majority of participants said that monkeys were closely related to humans.

## Conclusion

The present study suggested that farmers living in the forest buffer zones of Tamil Nadu were highly tolerant for HMC followed by HEC, HGC, and HWPC. The result of this study clearly showed that the farmers affected with HPC need to be sensitized for the conservation of this species despite its conflict behavior by suitable mitigation technologies.

Mere reduction in the incidence of crop damage through temporary means will not serve the purpose in the longrun. In general, wild animals are not much dangerous, until disturbed. This legitimate truth should be instilled into the minds of people in the buffer zone, through proper education and outreach, which would be the strategy for resolving conflict in these zones.

## Authors’ Contributions

This study is the part of the Ph.D thesis of the first author KS, who carried out the research under the guidance of Professor PM. CM and SG helped during the survey. The article was drafted by KS. The revision was made by PM, CM, SG, and MGJ. All authors have read and approved the final version of the manuscript.
